# Giant choledochal cyst type 4A: a surgical challenge

**DOI:** 10.11604/pamj.2020.37.95.24811

**Published:** 2020-09-25

**Authors:** Sakthivel Harikrishnan, Servarayan Murugesan Chandramohan, Apsara Chandramohan

**Affiliations:** 1 Surgical Gastroenterology, Government Stanley Medical College, Chennai, India,; 2 International Students Program, Sri Ramachandra Institute of Higher Education and Research, Chennai, India,; 3 Kilpauk Medical College, Chennai, India

**Keywords:** Choledochal cyst, giant choledochal cyst, choledochal cyst type 4A

## Abstract

Choledochal cysts are rare congenital anomalies of the biliary tract. There are 5 subtypes of choledochal cyst and in adults type 4 is the most common. Choledochal cyst rarely exceeds the size of 6cm in the reported literature. Only a handful of cases of giant choledochal cysts are reported in the literature. Here we report a case of a 33-year-old lady who presented with a huge abdominal mass and on evaluation was found to have a giant choledochal cyst type 4A. The patient underwent excision of the extrahepatic hugely dilated choledochal cyst with cholecystectomy and reconstruction was done by Roux-en-Y hepaticojejunostomy to the confluence of the right and left hepatic duct at the hilum.

## Introduction

Choledochal cysts are congenital malformation of the bile duct. There are 5 subtypes of choledochal cyst as described by Todanis classification. Type 1 is the most common in children and type 4 is the most common in adults. Giant choledochal cysts are defined as a cyst with a diameter of more than 10cm. Here we report a rare case of giant choledochal cyst and its successful surgical management.

## Patient and observation

**Figure 1:**
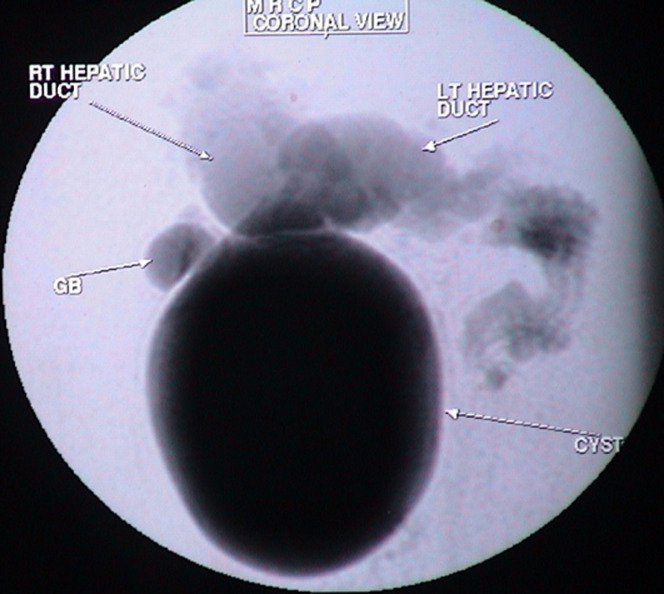
MRCP image showing massive cystic dilatation of the extrahepatic common hepatic duct and common bile duct (CBD) till the distal CBD with dilatation of the intrahepatic left and right hepatic duct

**Figure 2:**
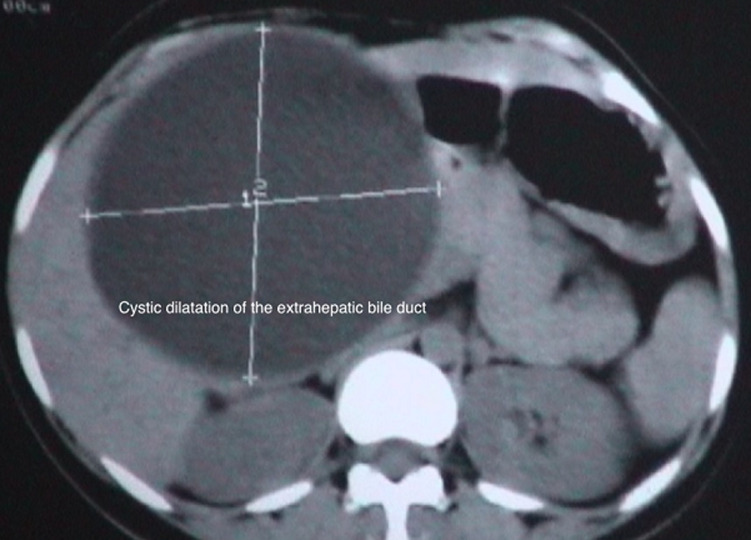
plain CT abdomen showing a cystic space-occupying lesion in the region of the head of the pancreas displacing the transverse colon and extending till the subhepatic space

**Figure 3:**
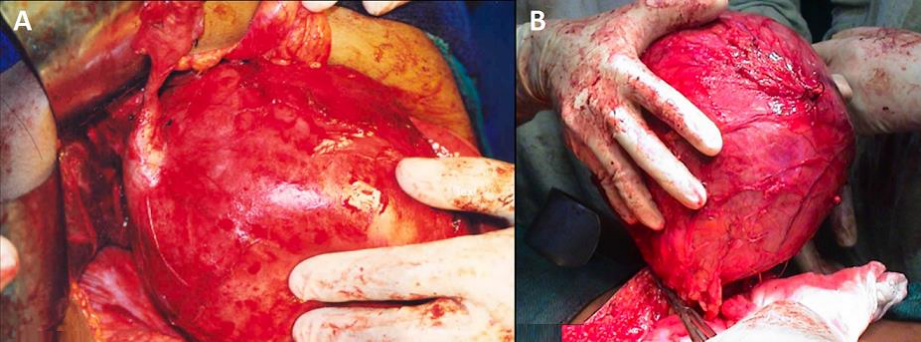
A) intraoperative picture showing the giant choledochal cyst dissected free of the surrounding structures; B) intraoperative picture showing the giant choledochal cyst being lifted after transecting the proximal end at the level of porta

A 30-year-old lady with no comorbid illness presented with complaints of swelling in the abdomen gradually increasing in size for 2 years and postprandial fullness. There was no history of sudden increase in the size of the abdominal lump or jaundice or fever with chills and rigors. The patient had mild dragging type of pain in the right upper abdomen with no specific aggravating or relieving factors. On examination, the patient was afebrile and anicteric. There was a firm mass with rounded borders measuring around 23cm in a horizontal dimension and 15cm in vertical dimension in the right hypochondrium extending into the right lumbar region and moving freely with respiration. The mass was continuous with the liver dullness and had a dull note on percussion. Blood investigations revealed a normal liver function test (Total bilirubin/direct bilirubin - 0.8/0.2, AST/ALT/SAP - 24/25/113, T. protein/s. albumin - 7.5/3.6). Imaging (MRI abdomen) showed a massive dilatation of the extrahepatic bile duct till the intrapancreatic portion measuring 23cm x 15cm suggestive of choledochal cyst. There was also dilatation of right, left hepatic duct and intrahepatic biliary radicals suggestive of a type 4A choledochal cyst (Figure 1, Figure 2). The patient underwent laparotomy by a midline incision and there was a giant choledochal cyst (massive globular dilatation of extrahepatic bile duct) measuring 23 x 15cm with dilatation of intrahepatic bile ducts. Gall bladder was collapsed. Because of the large choledochal cyst with imminent obstructive symptoms, the extrahepatic massively dilated bile duct was excised and reconstruction was done by Roux-en-Y hepaticojejunostomy. Proximally the choledochal cyst was transected at the level of hilum where there was an abrupt change in caliber and distally it was transected in the intrapancreatic waist of the cyst without damaging the pancreatic duct (Figure 3). Excised choledochal cyst was sent for histopathological examination and showed no evidence of malignancy.

## Discussion

Todanis classification divides choledochal cysts into 5 subtypes. Type 1 is limited to extrahepatic CHD and CBD. It is further subdivided into type 1a - cystic (the common type), type 1b - segmental and type 1c - fusiform/cylindrical. Type 2 is saccular diverticulum in the extrahepatic bile duct, type 3 is choledochocoele, type 4a is multiple cysts at intra and extrahepatic bile duct, type 4b is multiple cysts in the extrahepatic bile duct and type 5 is multiple cystic dilatation of intrahepatic biliary tree which can be focal or diffuse (carolis disease) [1]. Additional subtypes are type 1d - dilatation of cystic duct in addition to dilated CHD and CBD and type 6 - isolated dilation of the cystic duct [2]. The commonest type in all age groups is type 1 whereas in adults type 4 is more prevalent [3]. There are no definite size criteria to qualify for a choledochal cyst and usually, they do not exceed 6cm [4]. Giant choledochal cyst refers to a cyst size greater than 10cm [5]. The classic presentation of choledochal cyst like right upper quadrant pain, jaundice and abdominal mass is rarely seen in adults. Large choledochal cysts can lead to complications if left untreated. The excision of the extrahepatic component is the preferred treatment for type 4 choledochal cyst. The management of the intrahepatic portion of type 4 choledochal is controversial. Few groups have advocated minor hepatectomy like left lobectomy if confined to the left lobe. The risk of malignancy in the left out intrahepatic dilated duct after excision of the extrahepatic portion in type 4A cyst is negligible when compared to no treatment (45%) [6]. Only a few reports of giant choledochal cyst [5, 7-9] have been reported in the literature (Table 1).

**Table 1:** published literature on large choledochal cysts

Author/year of publication	Number of patients	Presentation	Type	Size in centimetres(cm)
Andrew J Holland *et al*./1996	1	Classic triad of mass, jaundice and pain	Type 1	30 cm
Anand *et al*./2013	10	Lump: 6 patients, cholangitis: 4 patients	Type 1: 6 patients, type 4: 4 patients	Mean: 14.2cm, range: 12 - 20cm
Nursel Yurttutan /2016	1	Jaundice/abdominal distension	Type 1	16cm
William J Farrel/1959	1	Pregnant lady with pain abdomen/jaundice	Type 1	Volume: 2600 cu.cm^*^

* cu.cm: cubic centimeters

## Conclusion

We report this very rare case of giant choledochal cyst type 4A measuring 23cm x 15cm and its successful excision of the extrahepatic component without decompressing/deroofing the cyst. Also, to the best of our knowledge, this is the largest reported type 4A choledochal cyst available in the literature.
